# Efficacy of metoclopramide for the treatment of acute migraine

**DOI:** 10.1097/MD.0000000000017065

**Published:** 2019-09-13

**Authors:** Chao Jiang, Ting Wang, Zheng-guo Qiu, Bo Chen, Bang-jiang Fang

**Affiliations:** a The Third Department of Neurology, The Second Affiliated Hospital of Xi’an Medical University, Xi’an; b Department of Emergency, Longhua Hospital Shanghai University of Traditional Chinese Medicine, Shanghai; c School of Economics and Management, Xi Dian University, Xi’an; d Department of Anesthesiology, The Second Affiliated Hospital of Xi’an Medical University; e Department of Anesthesiology, The Hospital of Xidian Group, Xi’an, Shaanxi, China.

**Keywords:** acute migraine, efficacy, metoclopramide, randomized controlled trial, safety

## Abstract

**Background:**

In this study, we will assess the efficacy and safety of metoclopramide for the treatment of acute migraine (AM).

**Methods:**

We will comprehensively search Cochrane Library, PUMBED, EMBASE, Google Scholar, Web of Science, Allied and Complementary Medicine Database, Chinese Biomedical Literature Database, and China National Knowledge Infrastructure from the inception to July 1, 2019 to identify any eligible studies. Only randomized controlled trials will be considered for inclusion. The study selection, data collection, and management will be completed by two authors independently. The risk of bias will be assessed using Cochrane risk of bias tool. RevMan 5.3 software will be used for statistical analysis.

**Results:**

The primary outcome includes pain intensity, as measured by visual analogue scale or others. The secondary outcomes are success rate, requirement of rescue medicine, quality of life, relapse, and adverse events.

**Conclusions:**

This study will summarize the latest evidence for the clinical efficacy and safety of metoclopramide for the treatment of AM.

**PROSPERO registration number:**

PROSPERO CRD42019142795.

## 1 Introduction

Acute migraine (AM) is a very common neurovascular disorder.^[1,2]^ It is characterized by a moderate to severe, recurrent, unilateral or bilateral, and headache which often lasts several hours to days.^[3–5]^ At the same time, people who experience this disorder often accompany a series of complications, such as nausea, vomiting, sensitivity to the light, sound, touch, or smell.^[6–8]^ Additionally, about 25% patients with AM also experience motor symptoms, transient visual or language disturbance.^[9,10]^ Unfortunately, its pathophysiology is still complex and insufficient understood. Therefore, if such disorder cannot be treated very well, it greatly affects health-related quality of life in patients with AM.^[11,12]^

Several previous clinical studies have reported that metoclopramide can mange AM effectively.^[13–23]^ However, no study has assessed its efficacy and safety systematically. Thus, this study will aim to systematically evaluate the efficacy and safety of metoclopramide for the treatment of patients with AM.

## 2 Methods

### 2.1 Criteria for including studies

#### 2.1.1 Types of studies

We will include randomized controlled trials (RCTs) of metoclopramide for AM regardless of language and publication status.

### 2.2 Types of interventions

The patients in the treatment group have received metoclopramide monotherapy alone.

The patients in the control group can receive any interventions, except metoclopramide.

### 2.3 Types of patients

Participants diagnosed with AM will be included with no limitation of race, gender, and economic status.

### 2.4 Types of outcome measurements

The primary outcome includes pain intensity, as measured by visual analogue scale or others. The secondary outcomes are success rate, requirement of rescue medicine, quality of life (it can be assessed by The Short Form-36 Health Survey or any relevant scales), relapse, and adverse events.

### 2.5 Search strategy

We will comprehensively search the following electronic databases from inception to the July 1, 2019: Cochrane Library, PUMBED, EMBASE, Google Scholar, Web of Science, Allied and Complementary Medicine Database, Chinese Biomedical Literature Database, and China National Knowledge Infrastructure. No language or publication date is limited. The sample of search strategy for Cochrane Library is shown in Table 1. Similar search strategies for other electronic databases will also be built.

**Table 1:**
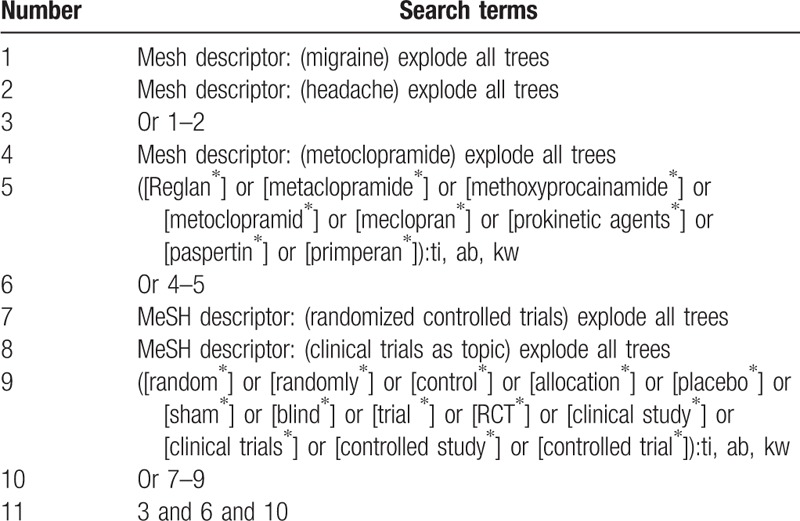
Search strategy for Cochrane Library.

In addition, any clinical registry, conference proceedings, reference lists of identified relevant RCTs, comments, and reviews will also be searched to identify any planned, ongoing, or unpublished literature.

### 2.6 Data collection and analysis

#### 2.6.1 Study selection

**Figure 1:**
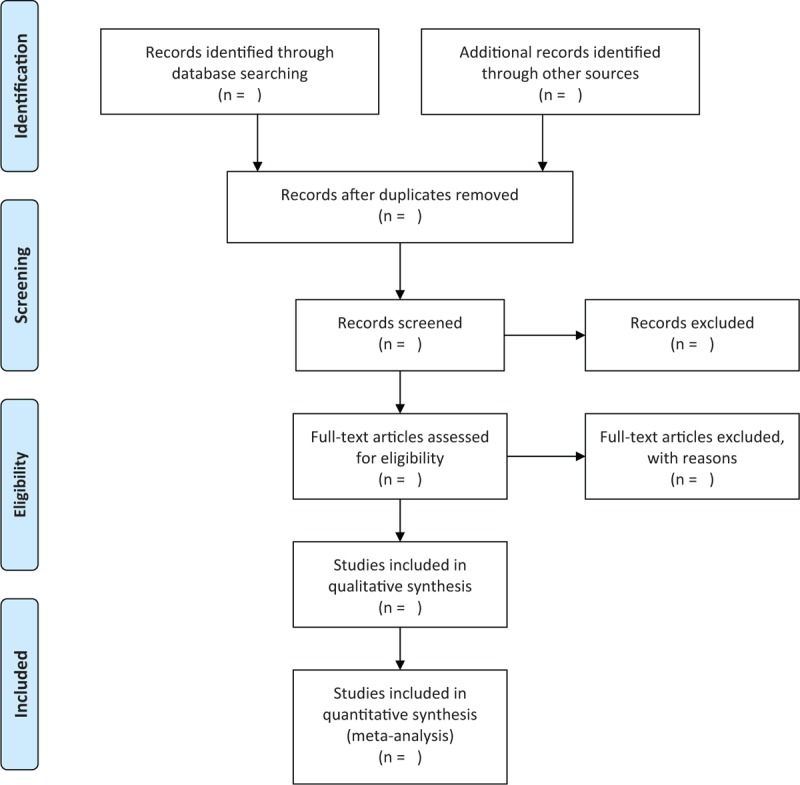
Flowchart of study selection.

The study selection will be completed by two authors independently. They will check all literature results each other. When any divergences occur, a third independent author will help to make the final decision by discussion. Full texts of all remaining potential studies will be read if it is necessary. Details of whole study selection process will be presented in the Preferred Reporting Items for Systematic Reviews and Meta-Analysis flowchart in Figure 1.

#### 2.6.2 Data extraction

A piloted data extraction form has been built by all authors. The following information will be collected from all eligible studies independently by two authors: general information of each included study, patient characteristics, study population, study setting, study methods, treatment details, outcome measurements, safety, and any others. Any different opinions will be resolved by discussion with the help of a third independent author.

#### 2.6.3 Missing data dealing with

If the data is unclear, missing or presented in the form that cannot be collected, we will attempt to contact primary authors by email to obtain those data if it is possible. If we cannot obtain those data from original authors, we will analyze available data only.

#### 2.6.4 Risk of bias assessment

Two authors will independently evaluate the risk of bias using Cochrane risk of bias tool. This tool comprises of 7 domains, and each one is further graded as high, low, or unclear risk of bias according to the relevant information extracted from each included study. Any different opinions will be solved by consensus with a third independent author.

#### 2.6.5 Methods of treatment measurements

For dichotomous data, risk ratio and 95% confidence intervals will be presented. For continuous data, mean difference or standardized mean difference, and 95% confidence intervals will be calculated.

#### 2.6.6 Assessment of heterogeneity

The heterogeneity among included studies will be tested by the *I*^2^ statistics. If the value of *I*^2^ is ≤50%, the heterogeneity will be minor, and a fixed-effect model will be applied. Otherwise, if the value of *I*^2^ is >50%, the heterogeneity across studies will be statistically substantial, and a random-effect model will be used.

#### 2.6.7 Assessment of reporting bias

We will check publication bias using funnel plot and Egger's regression test if >10 RCTs are included.^[24,25]^

### 2.7 Data synthesis

RevMan 5.3 software will be applied for statistical analysis. If the heterogeneity is minor (*I*^2^ ≤ 50%), we will apply a fixed-effect model for data pooling, and meta-analysis will be carried out. Otherwise, if the heterogeneity is substantial (*I*^2^ > 50%), we will use a random-effect model to pool the data, and subgroup analysis will be performed. A narrative description of the outcome results will be conducted when the meta-analysis is still not feasible after subgroup analysis.

#### 2.7.1 Subgroup analysis

We will carry out subgroup analysis to identify any possible causes of substantial heterogeneity if it is necessary. It will be carried out based on the type of treatments, comparators, and outcome measurements.

#### 2.7.2 Sensitivity analysis

We will perform sensitivity analysis to test the stability and robustness of pooled treatment effects by removing low quality studies.

## 3 Discussion

Metoclopramide may be used for the treatment of patients with AM. However, no study on this topic has been reported systematically. This study will summarize more convincing information on metoclopramide for the treatment of AM. In addition, its results will also provide information on the credibility current evidence and research directions for both clinical practice and further studies.

### 3.1 Ethics and dissemination

This study will not involve personal patient information, thus, no ethical approval will be required. The results of this study will be published in a peer-reviewed journal.

## Acknowledgment

The supporters had no role in this study.

## Author contributions

**Conceptualization:** Chao Jiang, Ting Wang, Bo Chen, Bang-jiang Fang.

**Data curation:** Chao Jiang, Zheng-guo Qiu, Bo Chen, Bang-jiang Fang.

**Formal analysis:** Chao Jiang, Ting Wang, Zheng-guo Qiu.

**Funding acquisition:** Bang-jiang Fang.

**Investigation:** Chao Jiang, Bang-jiang Fang.

**Methodology:** Chao Jiang, Ting Wang, Zheng-guo Qiu, Bo Chen.

**Project administration:** Bang-jiang Fang.

**Resources:** Chao Jiang, Ting Wang, Zheng-guo Qiu, Bo Chen, Bang-jiang Fang.

**Software:** Ting Wang, Zheng-guo Qiu, Bo Chen.

**Supervision:** Bang-jiang Fang.

**Validation:** Chao Jiang, Ting Wang, Bo Chen, Bang-jiang Fang.

**Visualization:** Chao Jiang, Zheng-guo Qiu, Bang-jiang Fang.

**Writing – original draft:** Chao Jiang, Ting Wang, Zheng-guo Qiu, Bo Chen, Bang-jiang Fang.

**Writing – review & editing:** Chao Jiang, Ting Wang, Zheng-guo Qiu, Bo Chen, Bang-jiang Fang.
